# Supplementary material for: Fast hierarchical Bayesian analysis of population structure
Source: Nucleic Acids Res. 2019 May 11;47(11):5539–49. doi: 10.1093/nar/gkz361 (PMC6582336; doi:10.1093/nar/gkz361)
Supplement: gkz361_Supplemental_File [file gkz361_supplemental_file.pdf]

Supplementary Material for Fast Hierarchical Bayesian Analysis of Population Structure

Gerry Tonkin-Hill[1], John A. Lees[2], Stephen D. Bentley[1], Simon D.W. Frost[3,4], and Jukka Corander[1,5,6]

[1]Parasites and Microbes, Wellcome Sanger Institute, Cambridge, United Kingdom
[2]Department of Microbiology, New York University School of Medicine, 10016, USA
[3]Department of Veterinary Medicine, University of Cambridge, Cambridge, United Kingdom
[4]The Alan Turing Institute, London, United Kingdom
[5]Department of Biostatistics, University of Oslo, Blindern, 0317, Norway
[6]Helsinki Institute for Information Technology HIIT, Department of Mathematics and Statistics, University of Helsinki, 00014 Finland

**Parameter**	**Value(s)**
Number of replicates	3
Number of demes (clusters)	5, 10, 15, 20, 25
Deme level population size	100
Sequence length	100,000
Mutation rate	0.01
Recombination rate	10, 100
Migration rate	0.001, 0.01, 0.02
Proportion of population sampled	0.1

Supplementary Table 1: Parameters used to simulate genetic population structure using scrm [1]

**Dataset**	**Number of sequences**	**Number of sites**	**Reference**
Escherichia coli	1508	241750	Kallonen et al. 2017
Haemophilus influenzae	75	113605	Koelman et al. 2017
Listeria monocytogenes	128	150759	Kremer et al. 2017
Neisseria meningitidis	882	87730	Lees et al. 2017
Staphylococcus aureus	284	50104	Aanensen et al. 2016
Streptococcus pneumoniae	3156	392524	Chewapreecha et al. 2014
Ebola	1610	2279	Dudas et al. 2017
HIV	118091	1497	http://www.hiv.lanl.gov

Supplementary Table 2: The datasets used in the comparison of different clustering methods. The HIV and pneumococcal datasets were only compared with k-means and fastbaps as they are too large for other model based methods.

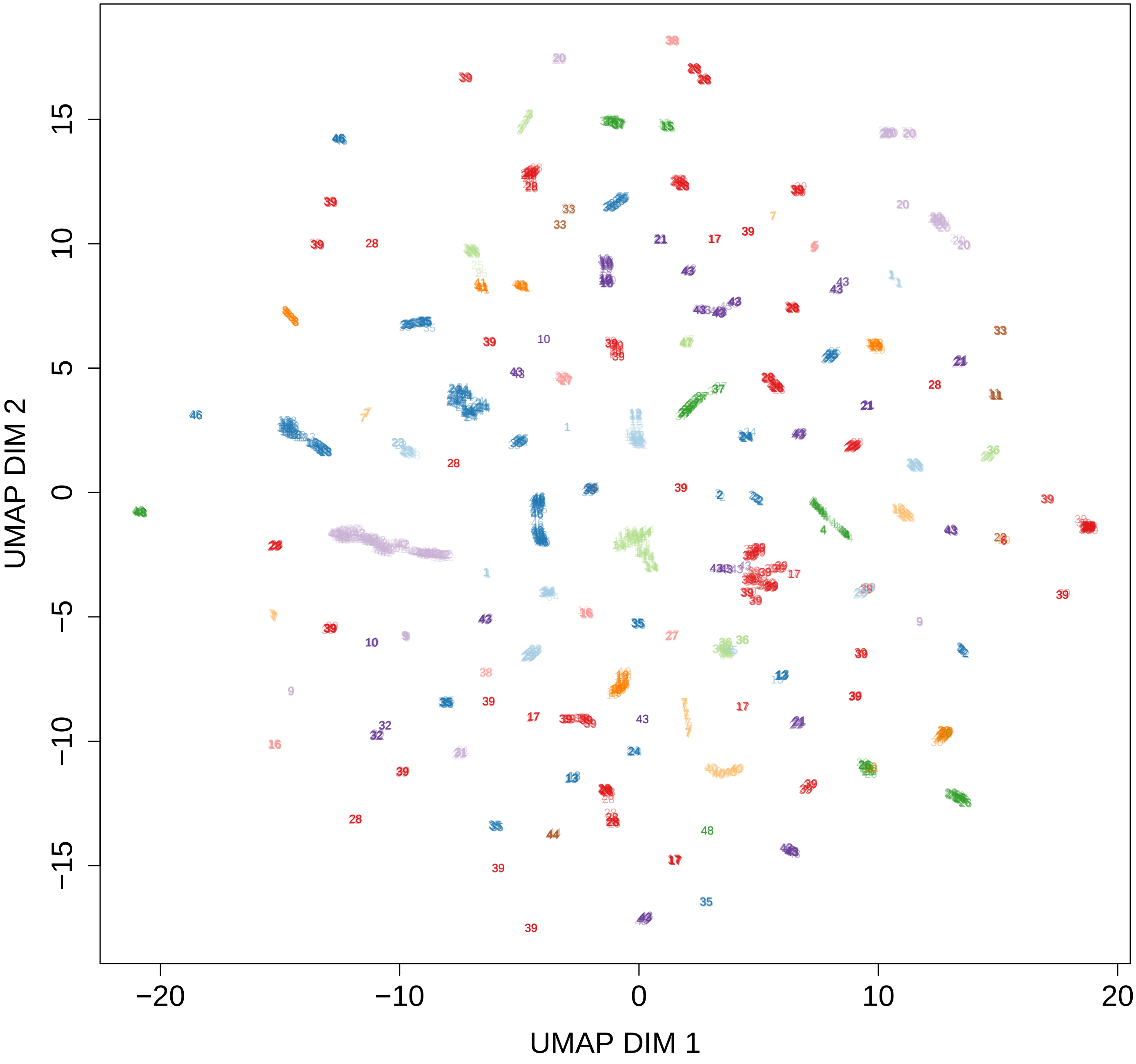

Supplementary Figure 1: A UMAP plot of Pneumococcal isolates coloured and numbered by their inferred clustering using k-means with the number of underlying clusters found using the elbow method ($k = 48$).

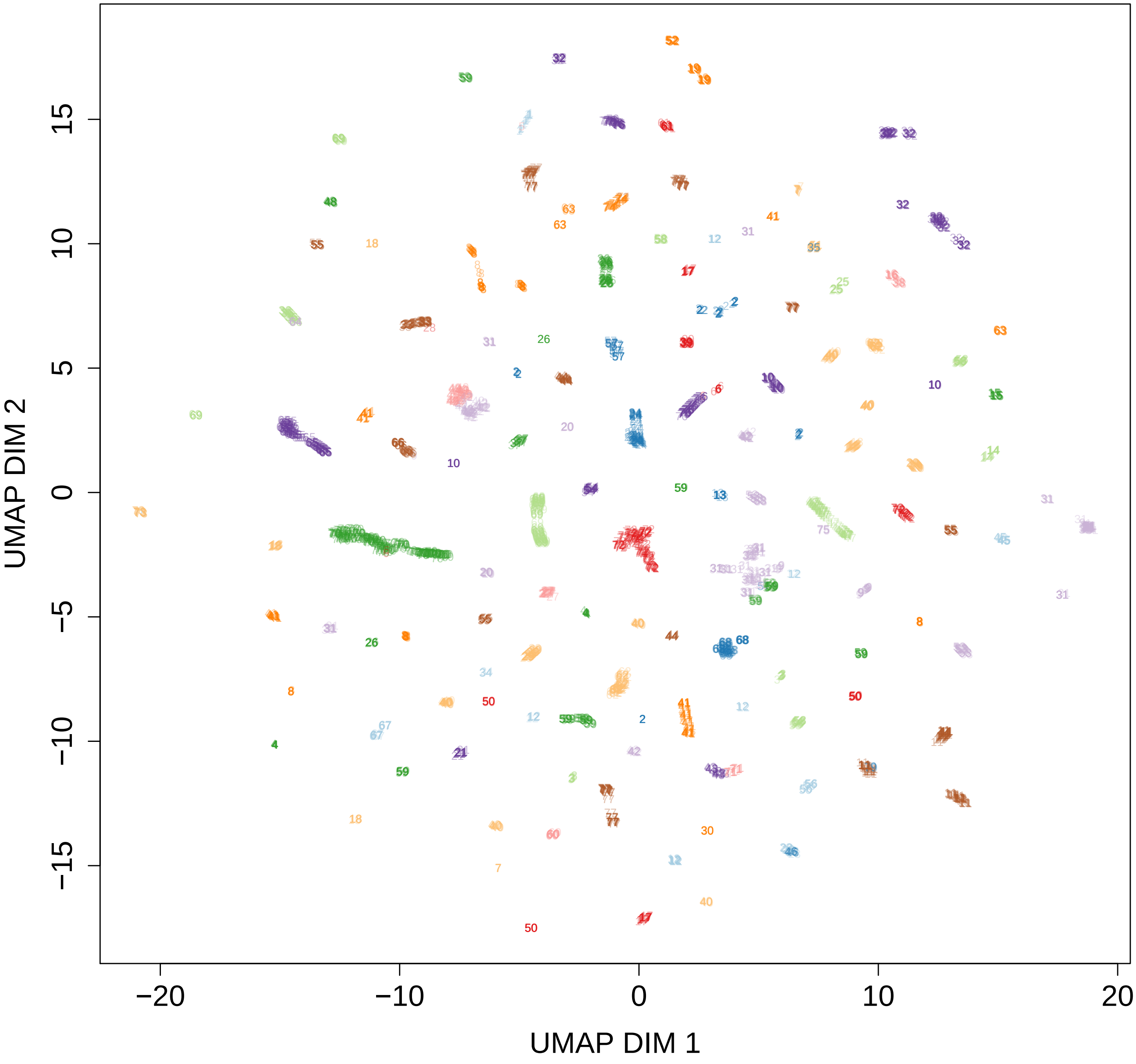

Supplementary Figure 2: A UMAP plot of Pneumococcal isolates coloured and numbered by their inferred clustering using k-means with the number of underlying clusters matching that found using fastbaps ($k = 79$)

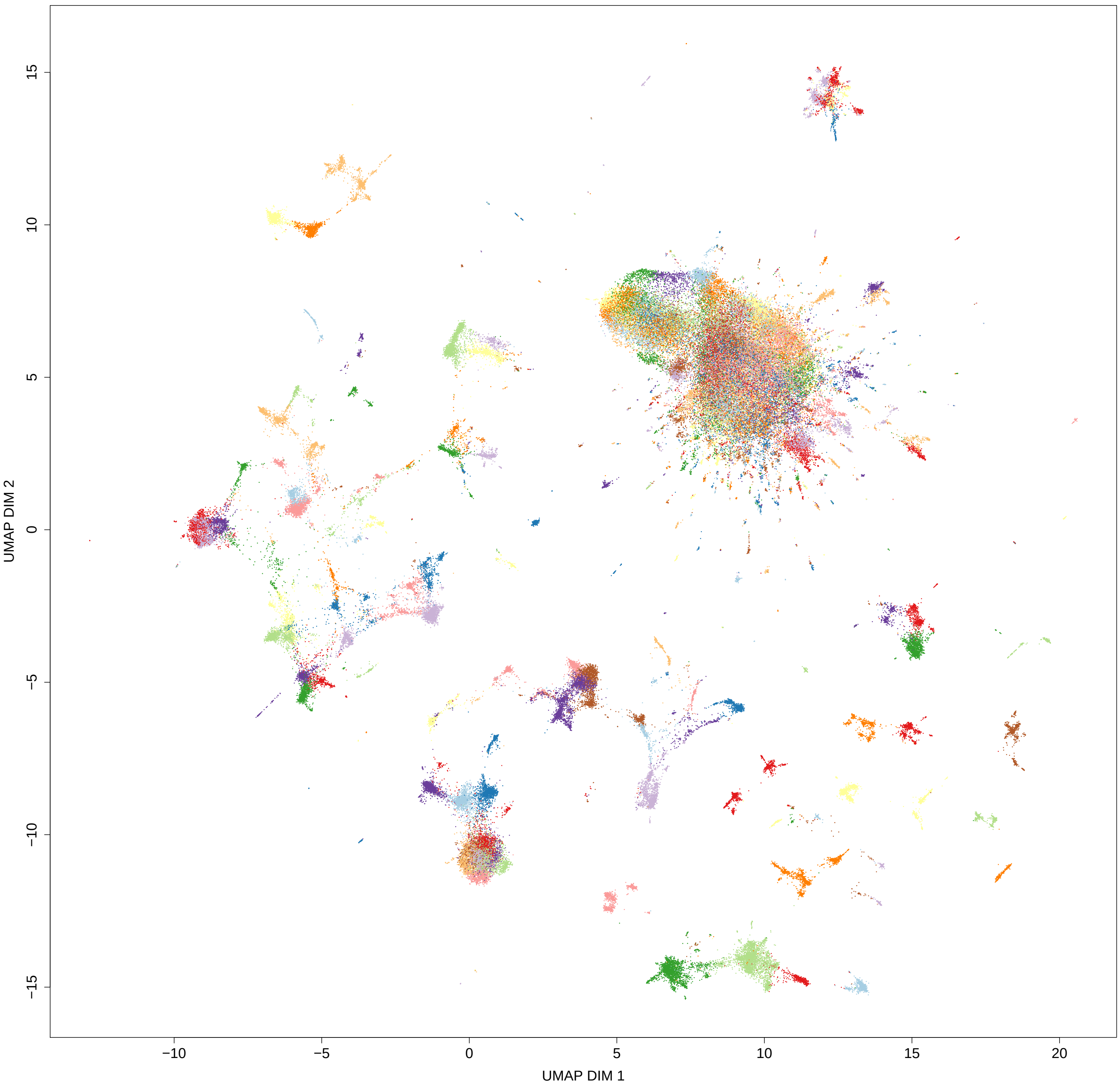

Supplementary Figure 3: A UMAP plot of HIV isolates coloured by their inferred clustering using k-means with the number of underlying clusters matching that found using fastbaps ($k = 193$)

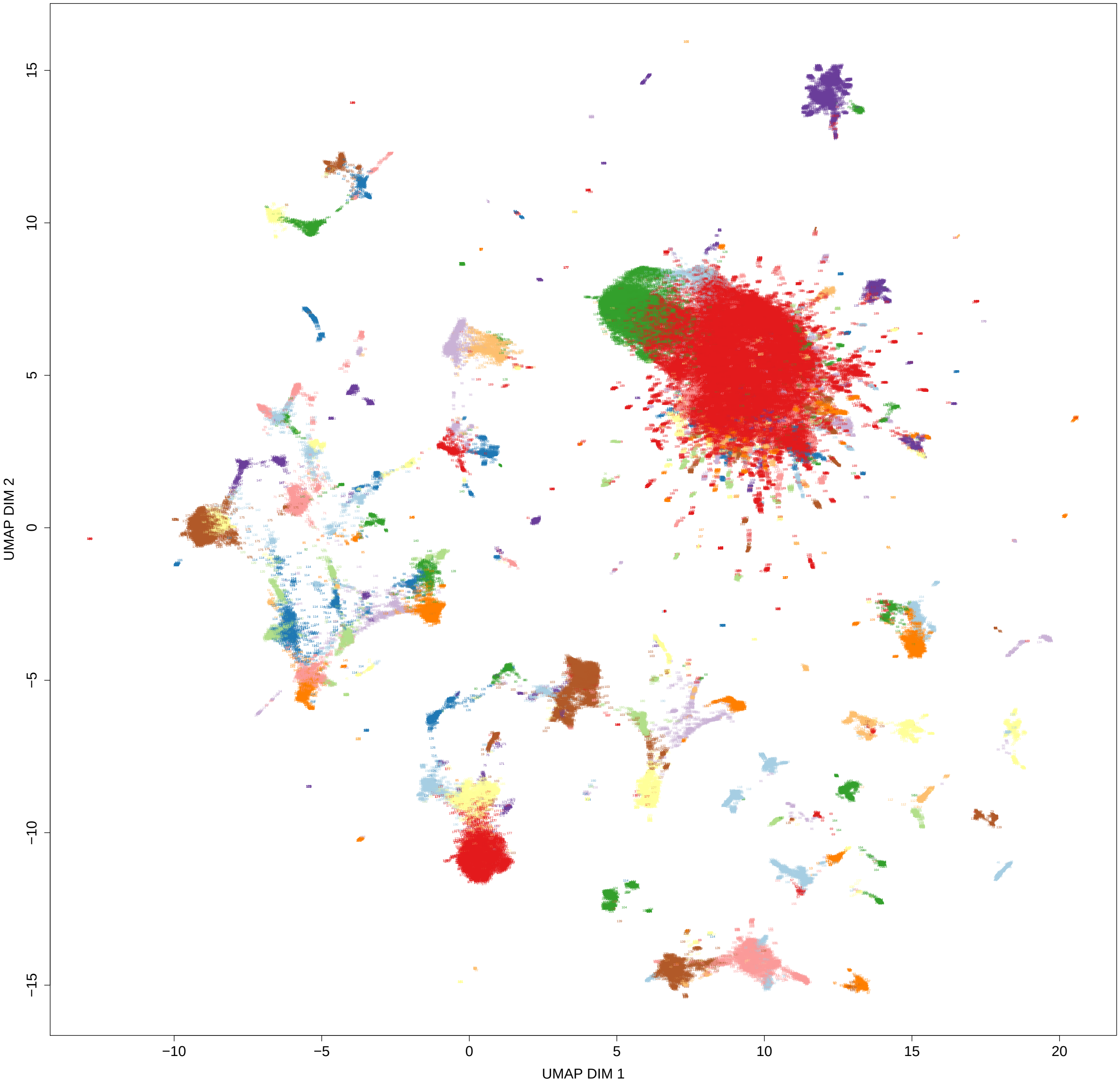

Supplementary Figure 4: A UMAP plot of HIV isolates coloured and numbered by their inferred clustering using fastbaps with the optimised BAPS prior ($k = 193$)

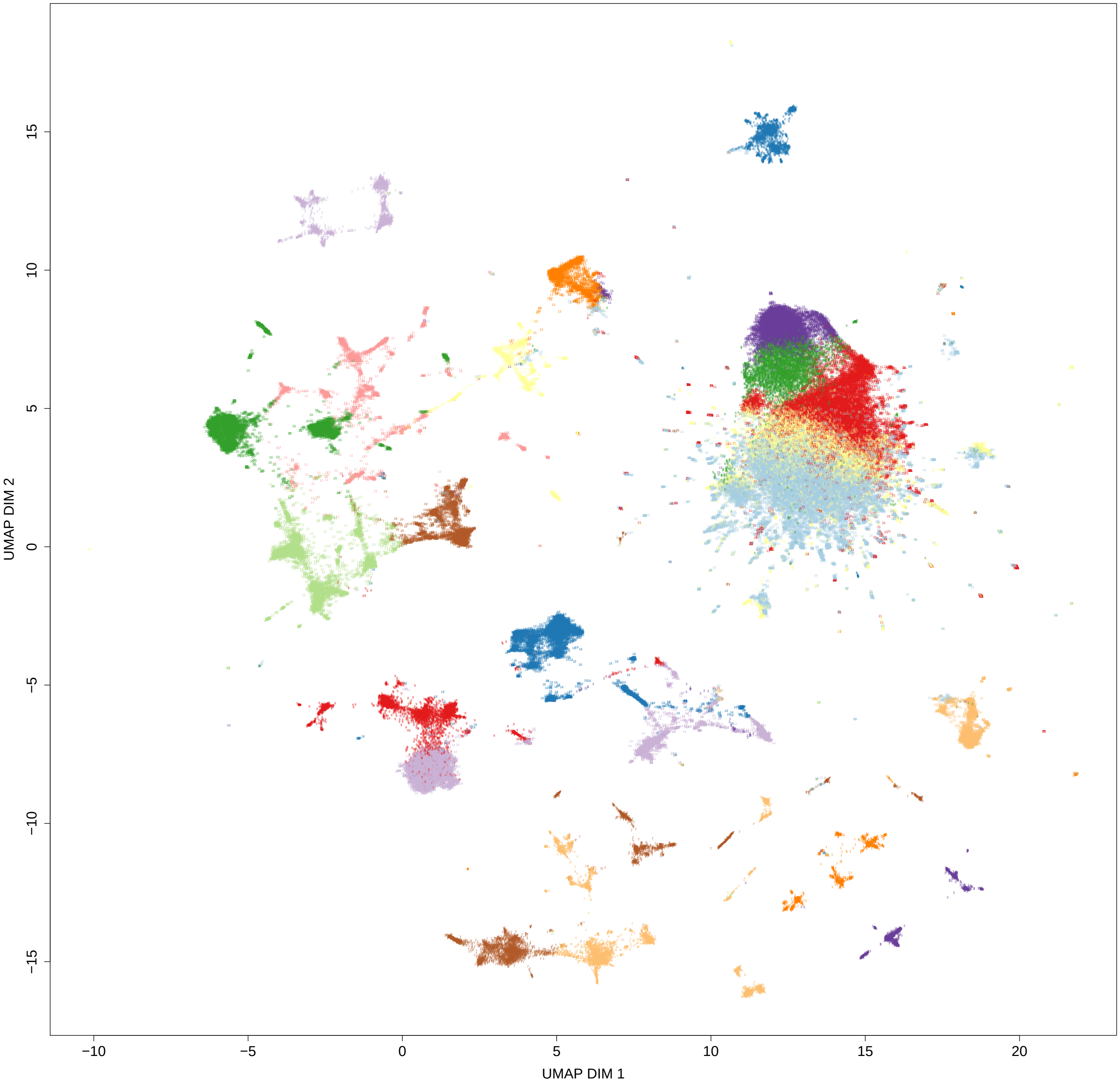

Supplementary Figure 5: A UMAP plot of HIV isolates coloured and numbered by their inferred clustering using k-means with the number of underlying clusters found using the elbow method ($k = 21$)

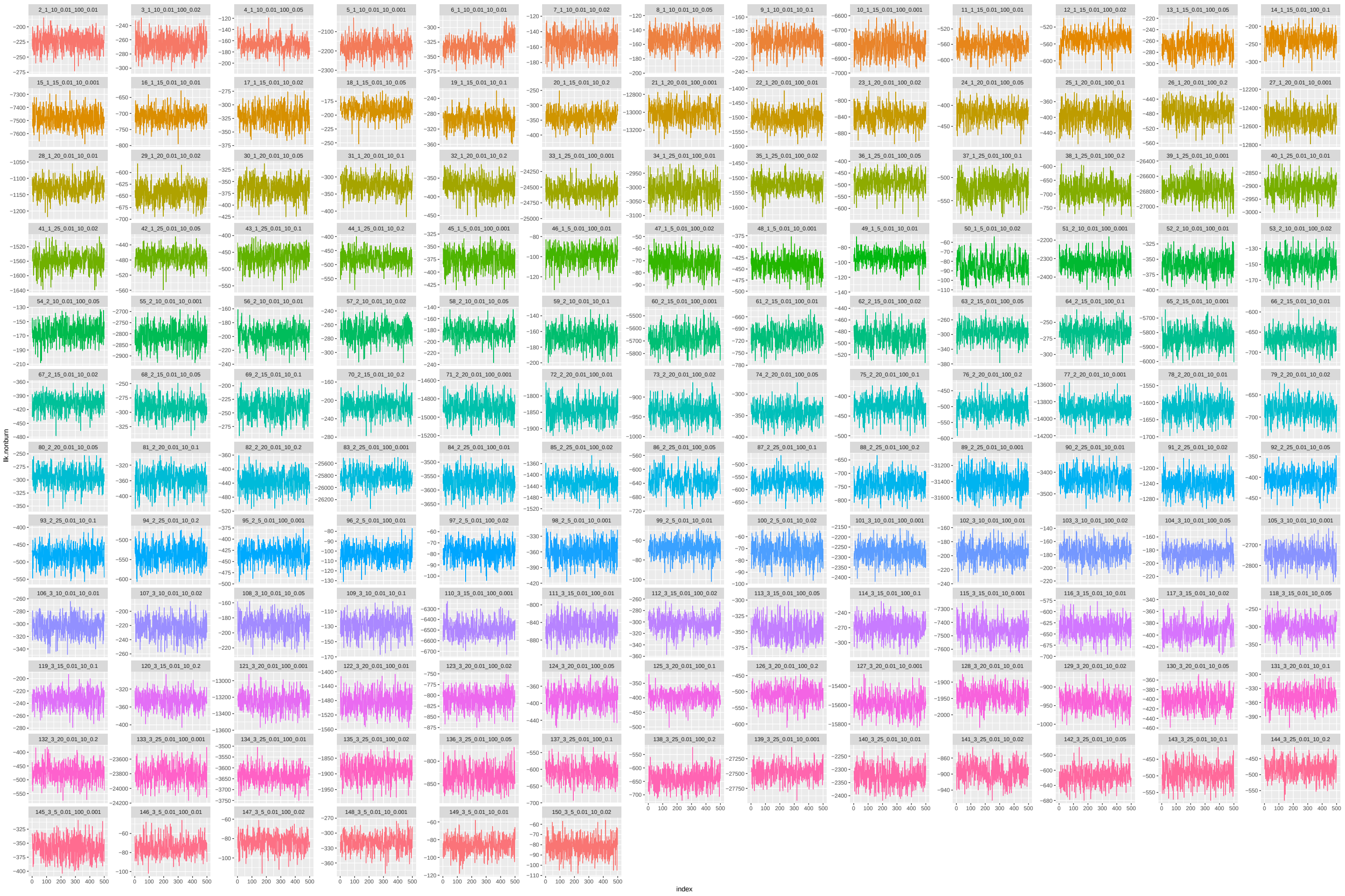

Supplementary Figure 6: The MCMC chains for each run of the STRUCTURE algorithm on the simulated datasets

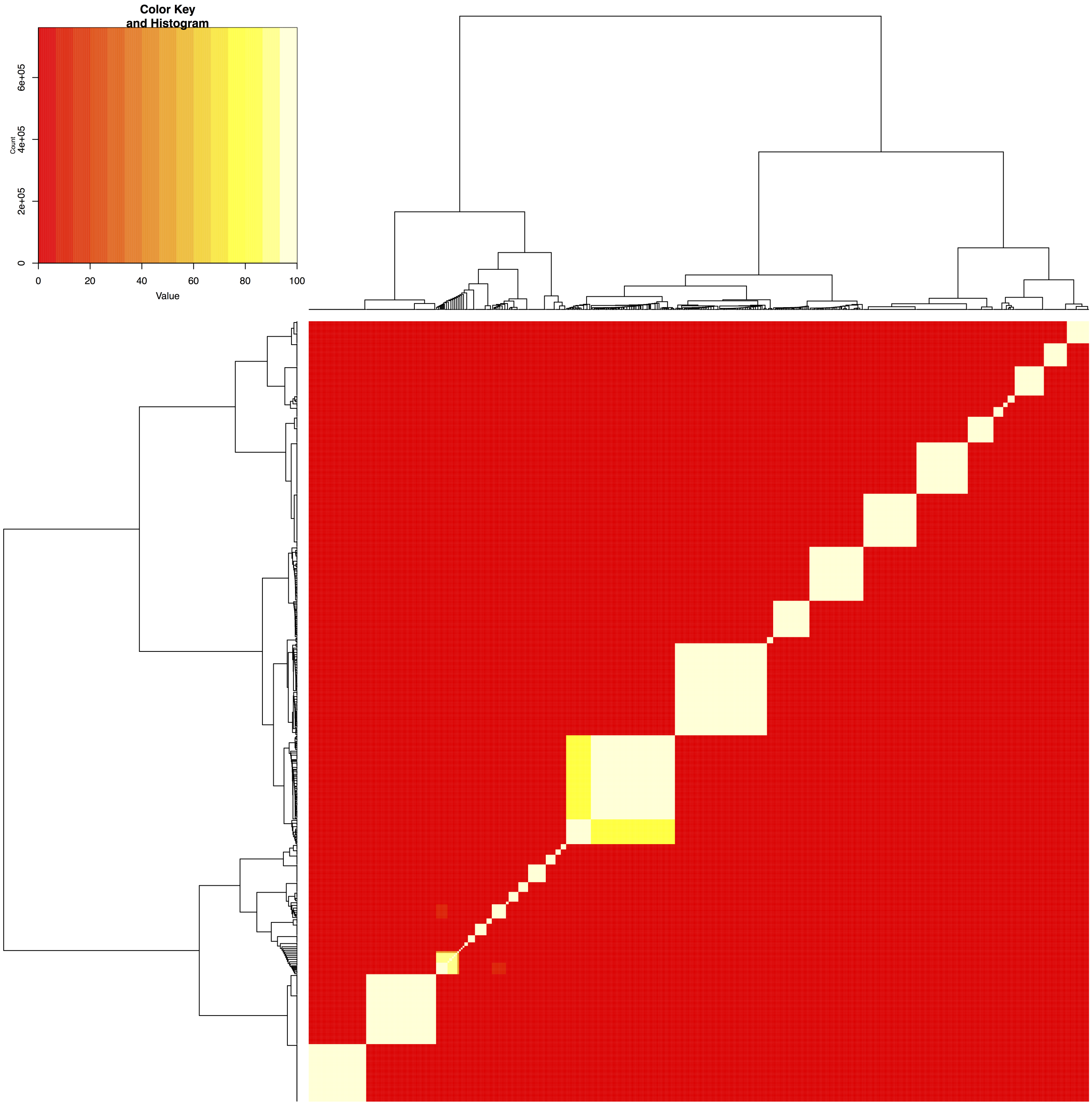

Supplementary Figure 7: A heatmap representing the bootstrap confidence of running fastbaps with the optimised BAPS prior on the *Neisseria meningitidis* dataset.

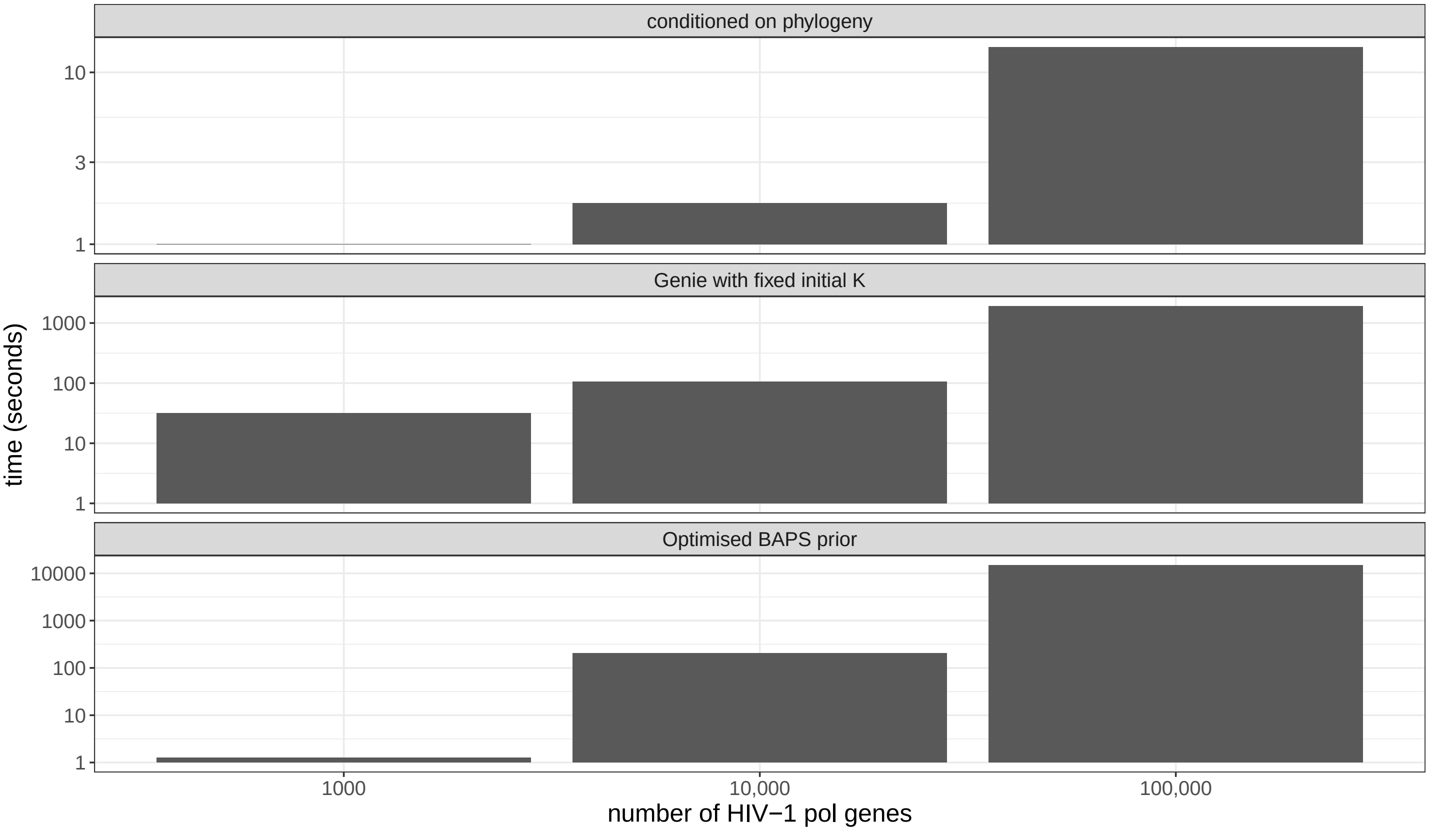

Supplementary Figure 8: A bar chart indicating the time complexity of the different modes of running fastbaps. The phylogeny conditioned algorithm scales linearly with number of samples whilst the full mode scales quadratically. If the initial number of clusters is fixed and a sub-quadratic initial clustering algorithm is used the method scales sub-quadratically as indicated by the Genie mode.

References

[1] Paul R Staab, Sha Zhu, Dirk Metzler, and Gerton Lunter. scrm: efficiently simulating long sequences using the approximated coalescent with recombination. *Bioinformatics*, 31(10):1680–1682, May 2015.

[2] T Kallonen, H J Brodrick, S R Harris, J Corander, and others. Systematic longitudinal survey of invasive escherichia coli in england demonstrates a stable population structure only transiently disturbed by the emergence of .... *Genome*, 2017.

[3] P H C Kremer, J A Lees, M M Koopmans, B Ferwerda, A W M Arends, M M Feller, K Schipper, M Valls Seron, A van der Ende, M C Brouwer, D van de Beek, and S D Bentley. Benzalkonium tolerance genes and outcome in listeria monocytogenes meningitis. *Clin. Microbiol. Infect.*, 23(4):265.e1–265.e7, April 2017.

[4] John A Lees, Philip H C Kremer, Ana S Manso, Nicholas J Croucher, Bart Ferwerda, Mercedes Valls Serón, Marco R Oggioni, Julian Parkhill, Matthijs C Brouwer, Arie van der Ende, Diederik van de Beek, and Stephen D Bentley. Large scale genomic analysis shows no evidence for pathogen adaptation between the blood and cerebrospinal fluid niches during bacterial meningitis. *Microb Genom*, 3(1):e000103, January 2017.

[5] David M Aanensen, Edward J Feil, Matthew T G Holden, Janina Dordel, Corin A Yeats, Artemij Fedosejev, Richard Goater, Santiago Castillo-Ramírez, Jukka Corander, Caroline Colijn, Monika A Chlebowicz, Leo Schouls, Max Heck, Gerlinde Pluister, Raymond Ruimy, Gunnar Kahlmeter, Jenny Åhman, Erika Matuschek, Alexander W Friedrich, Julian Parkhill, Stephen D Bentley, Brian G Spratt, Hajo Grundmann, and European SRL Working Group. Whole-Genome sequencing for routine pathogen surveillance in public health: a population snapshot of invasive staphylococcus aureus in europe. *MBio*, 7(3), May 2016.

[6] Claire Chewapreecha, Simon R Harris, Nicholas J Croucher, Claudia Turner, Pekka Marttinen, Lu Cheng, Alberto Pessia, David M Aanensen, Alison E Mather, Andrew J Page, Susannah J Salter, David Harris, Francois Nosten, David Goldblatt, Jukka Corander, Julian Parkhill, Paul Turner, and Stephen D Bentley. Dense genomic sampling identifies highways of pneumococcal recombination. *Nat. Genet.*, 46(3):305–309, March 2014.

[7] Gytis Dudas, Luiz Max Carvalho, Trevor Bedford, Andrew J Tatem, Guy Baele, Nuno R Faria, Daniel J Park, Jason T Ladner, Armando Arias, Danny Asogun, Filip Bielejec, Sarah L Caddy, Matthew Cotten, Jonathan D'Ambrozio, Simon Dellicour, Antonino Di Caro, Joseph W Diclaro, Sophie Duraffour, Michael J Elmore, Lawrence S Fakoli, Ousmane Faye, Merle L Gilbert, Sahr M Gevao, Stephen Gire, Adrianne Gladden-Young, Andreas Gnirke, Augustine Goba, Donald S Grant, Bart L Haagmans, Julian A Hiscox, Umaru Jah, Jeffrey R Kugelman, Di Liu, Jia Lu, Christine M Malboeuf, Suzanne Mate, David A Matthews, Christian B Matranga, Luke W Meredith, James Qu, Joshua Quick, Suzan D Pas, My V T Phan, Georgios Pollakis, Chantal B Reusken, Mariano Sanchez-Lockhart, Stephen F Schaffner, John S Schieffelin, Rachel S Sealfon, Etienne Simon-Loriere, Saskia L Smits, Kilian Stoecker, Lucy Thorne, Ekaete Alice Tobin, Mohamed A Vandi, Simon J Watson, Kendra West, Shannon Whitmer, Michael R Wiley, Sarah M Winnicki, Shirlee Wohl, Roman Wölfel, Nathan L Yozwiak, Kristian G Andersen, Sylvia O Blyden, Fatorma Bolay, Miles W Carroll, Bernice Dahn, Boubacar Diallo, Pierre Formenty, Christophe Fraser, George F Gao, Robert F Garry, Ian Goodfellow, Stephan Günther, Christian T Happi, Edward C Holmes, Brima Kargbo, Sakoba Keïta, Paul Kellam, Marion P G Koopmans, Jens H Kuhn, Nicholas J Loman, N'faly Magassouba, Dhamari Naidoo, Stuart T Nichol, Tolbert Nyenswah, Gustavo Palacios, Oliver G Pybus, Pardis C Sabeti, Amadou Sall, Ute Ströher, Isatta Wurie, Marc A Suchard, Philippe Lemey, and Andrew Rambaut. Virus genomes reveal factors that spread and sustained the ebola epidemic. *Nature*, 544(7650):309–315, April 2017.

[8] Los Alamos National Laboratory. HIV databases. `https://www.hiv.lanl.gov/content/index`. Accessed: 2018-10-25.
